# Mid-trimester Cervico-Isthmic Pregnancy With Placenta Accreta Spectrum Mimicking Placenta Previa and Cesarean Scar Pregnancy: A Diagnostic Imaging Pitfall

**DOI:** 10.7759/cureus.111514

**Published:** 2026-06-25

**Authors:** Masayoshi Komeda, Yutaka Iwagoi, Yasuhiro Yamamoto, Jun Sakata, Munekage Yamaguchi, Eiji Kondoh

**Affiliations:** 1 Obstetrics and Gynecology, Kumamoto University, Kumamoto, JPN

**Keywords:** cervico-isthmic pregnancy, cesarean scar pregnancy, diagnostic pitfall, placenta accreta spectrum, placenta previa

## Abstract

Cervico-isthmic pregnancy is a rare form of lower uterine implantation associated with a high risk of massive hemorrhage and placenta accreta spectrum (PAS), particularly when diagnosis is delayed. We report a woman in her 30s with a history of cesarean delivery who was referred at 19 weeks’ gestation with recurrent vaginal bleeding; no first-trimester imaging had been performed. Initial ultrasonography suggested marginal placenta previa, whereas subsequent magnetic resonance imaging (MRI) raised concern for cesarean scar pregnancy (CSP). However, careful imaging reassessment revealed misidentification of the cervical canal, with marked shortening of the true cervix and distension of the lower uterine segment, leading to a diagnosis of cervico-isthmic pregnancy. At 20 weeks, the patient developed a sudden massive hemorrhage requiring emergency hysterectomy. Histopathology confirmed cervical stromal implantation with PAS. This case highlights the diagnostic pitfalls of mid-trimester lower uterine implantation disorders and underscores the importance of early imaging and careful reassessment of the cervical canal.

## Introduction

Cervico-isthmic pregnancy is a rare form of lower uterine implantation associated with a high risk of massive hemorrhage and placenta accreta spectrum (PAS) [1-5]. In contemporary practice, most lower uterine implantation disorders are diagnosed in the first trimester owing to the widespread use of transvaginal ultrasonography, and advanced cases have become rare [2]. However, when early imaging is unavailable, diagnosis in the mid-trimester can be challenging. In women with a history of cesarean delivery, these conditions may mimic placenta previa or cesarean scar pregnancy (CSP), complicating accurate diagnosis and management.

We report a case of cervico-isthmic pregnancy in which the absence of first-trimester imaging and misidentification of the residual cervical canal resulted in delayed diagnosis. This case highlights the importance of early imaging and accurate identification of the cervical canal to avoid diagnostic error and guide appropriate management.

## Case presentation

A woman in her 30s (gravida 4, para 1) was referred to our hospital at 19 weeks and 5 days of gestation with recurrent vaginal bleeding and suspected marginal placenta previa. Her obstetric history included one prior cesarean delivery and two previous missed abortions managed by surgical evacuation.

The current pregnancy had not been evaluated in the first trimester. At 14 weeks and 6 days, transvaginal ultrasonography at a local clinic demonstrated a viable intrauterine pregnancy with the placenta located near the internal cervical os, and marginal placenta previa was suspected (Figure 1A).

**Figure 1:**
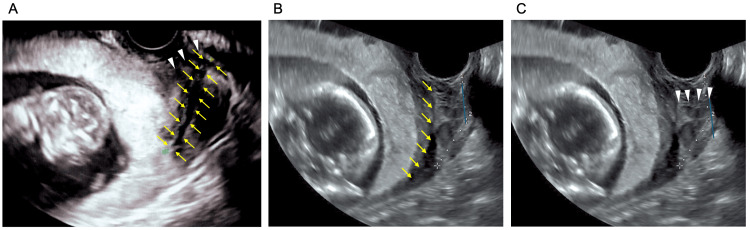
Transvaginal ultrasonography (A) Fourteen weeks of gestation (referring clinic). The placenta is located near the presumed internal cervical os. Yellow arrows indicate the cervical canal as initially interpreted, with apparently preserved cervical length. White arrowheads indicate the cervical canal location identified on reassessment. (B) Nineteen weeks of gestation (our institution). The structure marked by yellow arrows was initially interpreted as the cervical canal. (C) Reassessment of the 19-week image. White arrowheads indicate the true cervical canal, demonstrating marked shortening (approximately 1 cm).

After referral from the local clinic, she was admitted to a regional perinatal center because of recurrent vaginal bleeding. To further assess the implantation site and investigate the cause of the persistent bleeding, pelvic magnetic resonance imaging (MRI) was performed at 19 weeks and 4 days, demonstrating a nearly collapsed uterine corpus cavity (Figure 2A). The MRI findings raised concern for cesarean scar pregnancy, prompting referral to our tertiary university hospital for further evaluation and management.

**Figure 2:**
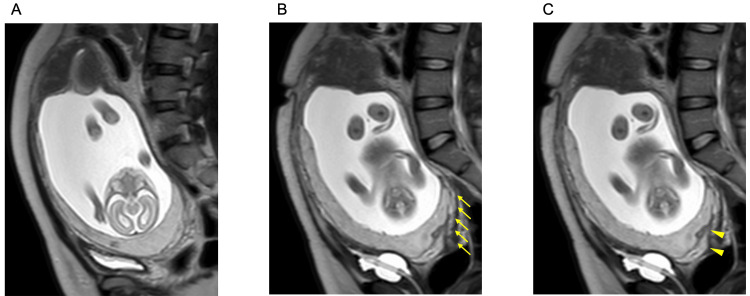
Pelvic magnetic resonance imaging at 19 weeks of gestation (A) Sagittal T2-weighted image demonstrating a nearly collapsed uterine corpus cavity. (B) Sagittal T2-weighted image showing the structure initially interpreted as the cervical canal (yellow arrow). (C) Reassessment of the image shown in (B). The presumed cervical canal corresponds to a vascular flow void within the posterior cervical lip. Yellow arrowheads indicate the cranial and caudal margins of the true cervical canal. Marked distension of the lower uterine segment is noted without focal anterior myometrial defect or bladder involvement.

On referral, the differential diagnosis included placenta previa, CSP, and cervico-isthmic pregnancy. MRI showed no focal anterior myometrial defect or bladder involvement, arguing against CSP. Instead, the lower uterine segment was markedly distended. Transvaginal ultrasonography demonstrated no increased vascularity between the placenta and bladder (data not shown) and no prominent placental lacunae. Careful reassessment of ultrasonography (Figure 1B, 1C) and MRI (Figure 2B, 2C) revealed that the structure initially interpreted as the cervical canal corresponded to a vascular flow void within the posterior cervical lip. The gestational sac and placenta appeared to involve both the cervix and lower uterine segment. The external cervical os remained closed on pelvic examination. The true residual cervical canal was markedly shortened to approximately 1 cm. These findings supported a diagnosis of cervico-isthmic pregnancy.

The patient was counselled regarding the high risk of catastrophic hemorrhage, the previable gestational age, and the substantial risk of hysterectomy. She chose to continue the pregnancy.

At 20 weeks of gestation, she developed sudden massive vaginal bleeding, and an emergency hysterotomy was performed as a life-saving intervention. At laparotomy, marked distension of the lower uterine segment was observed without gross evidence of bladder invasion. A 300 g stillborn fetus was delivered. The placenta was extensively adherent from the cervix to the lower uterine segment. Most of the placenta was forcibly removed manually, and balloon tamponade was attempted to achieve hemostasis and preserve the uterus; however, bleeding could not be controlled, necessitating total hysterectomy. Total blood loss was 4,800 mL.

Gross examination of the resected uterus demonstrated extensive placental attachment involving the cervix and lower uterine segment, extending beyond the presumed cesarean scar region and toward the vicinity of the external cervical os (Figure 3A). Histopathological examination demonstrated chorionic villi invading the cervical stroma, consistent with placenta increta (Figure 3B). The postoperative course was uneventful.

**Figure 3:**
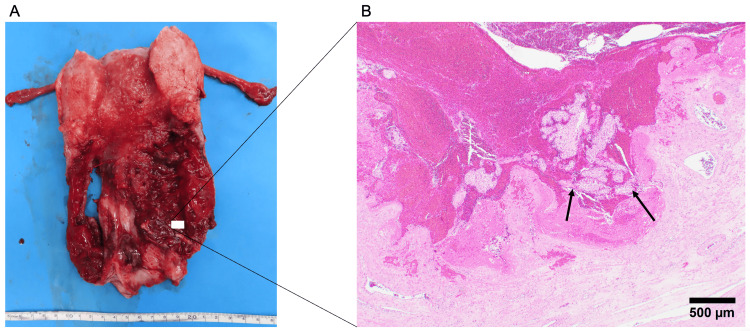
Macroscopic and histopathological findings (A) Gross appearance of the resected uterus demonstrating extensive placental attachment involving both the cervix and lower uterine segment, extending toward the vicinity of the external cervical os. (B) Histopathological examination (hematoxylin and eosin stain). Arrows indicate chorionic villi invading the cervical stroma without intervening decidua, consistent with placenta increta.

## Discussion

This case illustrates that cervico-isthmic pregnancy may remain unrecognized until the mid-trimester and can mimic placenta previa or CSP, particularly when early imaging is unavailable. Because cervico-isthmic pregnancy is frequently associated with PAS, delayed recognition may complicate surgical management and increase the risk of catastrophic hemorrhage. In our patient, recurrent bleeding prompted MRI at 19 weeks, yet the diagnosis remained uncertain until careful reassessment of imaging and subsequent histopathological confirmation.

Cervical pregnancy is classically defined as implantation below the internal cervical os within the endocervical canal. David et al. [1] proposed a spectrum of lower uterine implantation, including cervico-isthmic pregnancy, based on the primary site of placental attachment [2]. In this classification, cervico-isthmic pregnancy represents the upward extension of cervical implantation into the isthmic region. In the present case, histopathological examination demonstrated chorionic villi invading the cervical stroma, consistent with placenta increta, confirming cervical involvement. In addition, gross and imaging findings showed placental attachment involving both the cervix and lower uterine segment. Together, these pathological, gross, and imaging findings support a diagnosis of cervico-isthmic pregnancy.

The principal diagnostic challenge lay in the interpretation of imaging in the setting of markedly distorted cervico-isthmic anatomy. In the mid-trimester, ultrasonography and MRI suggested low anterior implantation in the lower uterine segment, raising suspicion for placenta previa or CSP. However, careful reassessment demonstrated that the structure initially interpreted as the cervical canal actually represented a vascular flow void within the posterior cervical lip. The misinterpretation primarily resulted from mistaking a vascular flow void within the posterior cervical lip for the cervical canal, while marked distension of the lower uterine segment and shortening of the true cervical canal likely further obscured the anatomical relationships. Previous reports have emphasized that the diagnosis of cervico-isthmic pregnancy depends on accurate identification of the cervical canal and the relationship between the gestational sac and uterine cavity [4,5].

Differentiation from CSP is clinically important because the anatomical basis of implantation influences surgical risk and management. CSP typically presents as focal implantation within a cesarean scar defect, often associated with anterior myometrial thinning and possible bladder involvement [6]. In contrast, cervico-isthmic pregnancy represents upward extension of a primary cervical implantation into the isthmic region and is not confined to a discrete cesarean scar defect [1,2,6,7]. In our case, the absence of a focal anterior defect, deep invasion, or bladder involvement on MRI, together with the lack of increased vascularity and placental lacunae on ultrasonography, argued against CSP and were more consistent with cervico-isthmic pregnancy.

From a management perspective, advanced cervico-isthmic pregnancy carries a substantial risk of catastrophic hemorrhage and frequently requires hysterectomy, particularly when complicated by PAS [1-5,8]. Although rare cases of progression to later gestation have been reported [1-3,5,8], such outcomes require highly specialized care and careful risk-benefit assessment. In this case, forcible placental removal in the setting of abnormal placental adherence resulted in uncontrolled hemorrhage, underscoring an important surgical principle: attempts at manual placental removal in PAS should be avoided. Following partial placental removal, intrauterine balloon tamponade was attempted as a rescue measure to control the ongoing hemorrhage. Although intrauterine balloon tamponade has been reported as a useful adjunct for hemorrhage control in selected patients with PAS, particularly during conservative management with the placenta largely left in situ [9], its effectiveness may be limited once extensive placental separation and massive hemorrhage have occurred. Therefore, management should prioritize rapid hemostasis and maternal stabilization, with hysterectomy often representing definitive treatment [10,11].

## Conclusions

Mid-trimester cervico-isthmic pregnancy may mimic placenta previa or CSP when early first-trimester imaging is unavailable. Recognition of altered cervico-isthmic anatomy and careful reassessment of the cervical canal are essential for accurate diagnosis and appropriate management. Given the strong association between cervico-isthmic pregnancy and PAS, early recognition is also critical for appropriate surgical planning and counseling because of the substantial risk of catastrophic hemorrhage and hysterectomy.
